# Attention biases the process of risky decision‐making: Evidence from eye‐tracking

**DOI:** 10.1002/pchj.724

**Published:** 2023-12-28

**Authors:** Mengchen Hu, Ruosong Chang, Xue Sui, Min Gao

**Affiliations:** ^1^ School of Psychology, Liaoning Collaborative Innovation Center of Children and Adolescents Healthy Personality Assessment and Cultivation Liaoning Normal University Dalian China

**Keywords:** attention, evidence accumulation, eye‐tracking, risk preference, risky decision‐making

## Abstract

Attention determines what kind of option information is processed during risky choices owing to the limitation of visual attention. This paper reviews research on the relationship between higher‐complexity risky decision‐making and attention as illustrated by eye‐tracking to explain the process of risky decision‐making by the effect of attention. We demonstrate this process from three stages: the pre‐phase guidance of options on attention, the process of attention being biased, and the impact of attention on final risk preference. We conclude that exogenous information can capture attention directly to salient options, thereby altering evidence accumulation. In particular, for multi‐attribute risky decision‐making, attentional advantages increase the weight of specific attributes, thus biasing risk preference in different directions. We highlight the significance of understanding how people use available information to weigh risks from an information‐processing perspective via process data.

## INTRODUCTION

Risky decision‐making involves trade‐offs between benefits and risks and occurs in all aspects of life. Risk preference is not fixed, and people will be risk‐seeking in some situations and risk‐averse in others (Barrafrem & Hausfeld, 2020; Clay et al., 2017). Researchers have long sought to clarify how people weigh risks and make decisions. Recent studies have aimed to explain the process behind preferential or consumer decision‐making through the attentional mechanism (Pachur et al., 2018; Stewart et al., 2016; Vriens et al., 2020; Wedel et al., 2022). Individuals tend to choose options that receive more attention (Gluth et al., 2020; Peschel et al., 2019; Robertson & Lunn, 2020; Smith & Krajbich, 2019). However, whether attention can explain the more complex process of risky decision‐making needs to be further explored.

Early decision theory, such as prospect theory, focused on describing people's final choice results (Kahneman, 1979; Tversky & Kahneman, 1992). For example, risk preference is a function of the expected value calculated from weighted outcomes and probabilities (Kahneman, 1979; Tversky & Kahneman, 1992). Subsequently, researchers set out to explore process‐based models; based on this exploration, dual process theory (DPT) was proposed. DPT holds that risky decision‐making results from competition between a fast, intuitive, automatic System 1 and a deliberate, conscious, controlled System 2 (Evans & Stanovich, 2013; Kahneman & Frederick, 2007; Metcalfe & Mischel, 1999; St Evans, 2008; Stanovich & West, 2000). More engagement in System 1 leads to heuristic decision‐making; otherwise, analytical decision‐making occurs in System 2 (Evans, 2006; Evans & Stanovich, 2013). Heuristic decision‐making simplifies the choice process and induces more irrational behavior, while the engagement of analytical decision‐making leads to more rational choices (Cui et al., 2022; Mukherjee, 2010; Roberts et al., 2021; Su et al., 2013).

However, researchers have pointed out that Systems 1 and 2 are a matter of degree and that there may be a single thought process to manage choices (Grayot, 2019; Keren & Schul, 2009; Krajbich et al., 2015). Psychoeconomic functions or DPT cannot provide a substantive explanation of the mechanism behind risky decision‐making. These theories assume that risky choices are made under high‐level cognitive processes, ignoring the premise that individuals choose in an environment in which relevant information is visually accessible (Orquin et al., 2021). The underlying cognitive mechanism involved in decision‐making depends heavily on acquiring and processing visual information (Gluth et al., 2020; Orquin et al., 2021; Wedel et al., 2022). Thus, visual attention may play a decisive role as a gatekeeper for incoming information (Duerrschmid & Danner, 2018; Orquin et al., 2018). For example, information search can explain amplified framing effects under time pressure, which were previously attributed to DPT (Roberts et al., 2021). Therefore, paying attention to the attentional process may highlight how risk preference is shaped.

In addition, many theories or models infer attention from outcome data, such as reaction time (RT) and choice results. However, multiple unobserved cognitive processes may lead to differences in endpoint measures (Wedel et al., 2022). Process metrics are critical to the predictive accuracy of risky decision‐making (Mueller et al., 2022). In this regard, eye‐tracking is considered a direct tool with which to identify and measure visual attention, allowing us to trace decision‐making without interrupting it and to infer how information input and processing occur (Ashby, Jekel, et al., 2016; Orquin et al., 2018; Rahal & Fiedler, 2019; Yoo et al., 2021). Eye‐tracking data can provide moderately accurate predictions about individual preferences (Ghaffari & Fiedler, 2018; Jonikaitis et al., 2017; Liu et al., 2020; Robertson & Lunn, 2020; Yoon et al., 2020). Among eye‐tracking metrics, fixation has been widely used in studies on the relationship between attention and decision‐making (Duerrschmid & Danner, 2018; Rahal & Fiedler, 2019). Fixation is a relatively long period of time during which a stimulus is held in the foveal region, typically lasting 200–500 ms (Rayner, 1998). Table 1 presents the eye‐tracking metrics related to fixation and the meanings they reveal.

**TABLE 1:** Definitions and meanings of fixation measures used in attention and decision‐making research (Duerrschmid & Danner, 2018; Rahal & Fiedler, 2019).

Sort	Measure	Definition	Meaning
Fixation duration	Individual fixation duration	Duration of each single fixation within an AOI	Processing depth and decision effort; weight of information
	Total fixation duration (relative dwell time)	Total duration across all fixations within an AOI	
Fixation count	Fixation count	Total number of fixations falling in an AOI	Search and processing extent
	Fixation likelihood (participants %)	Percentage of participants that have fixated at least once within an AOI	
Fixation sequence	First fixation	The position of the first fixation event within an AOI	Weight of information
	First fixation time	Start time of the first fixation to enter an AOI	
	Last fixation	The position of the last fixation event before a response being given within an AOI	
	Last fixation time	Start time of the last fixation to enter an AOI	

Abbreviation: AOI, area of interest.

In summary, attention affects the decision‐making process from the information‐processing perspective, and eye‐tracking provides a direct measurement. In light of this, this paper reviews the latest progress made in analysing the relationship between risky decision‐making and attention as illustrated by eye‐tracking, attempting to account for the process of risky decision‐making through the attentional mechanism. Although some reviews have focused on decision‐making and attention or eye movement in recent years, they have not focused on the specific domain of risky decision‐making (Motoki et al., 2021; Orquin et al., 2021; Wedel et al., 2022). Most previous studies on attention and decision‐making have focused on consumer choices (Ballco et al., 2019; Barbosa et al., 2021; Mehlhose et al., 2021; Tortora et al., 2019). Compared with consumer choices, risky decision‐making contains value information related to decision‐makers' benefits and involves risky trade‐offs, the visual stimuli of which are not as rich as those related to consumer goods (Dutilh & Rieskamp, 2016; Harrison & Swarthout, 2019; Zilker, 2022). Therefore, this focus is necessary as it specifies how visual attention acts with regard to more complex risky choices.

This paper intends to discuss the following three stages that can be used to explain the process of risky decision‐making under the effect of attention. How is attention before choices captured by option information? What happens to the process by which attention is biased? How does this process act on attribute information to change the resulting risk preference?

## ATTENTIONAL CAPTURE BEFORE RISKY CHOICES

**FIGURE 1:**
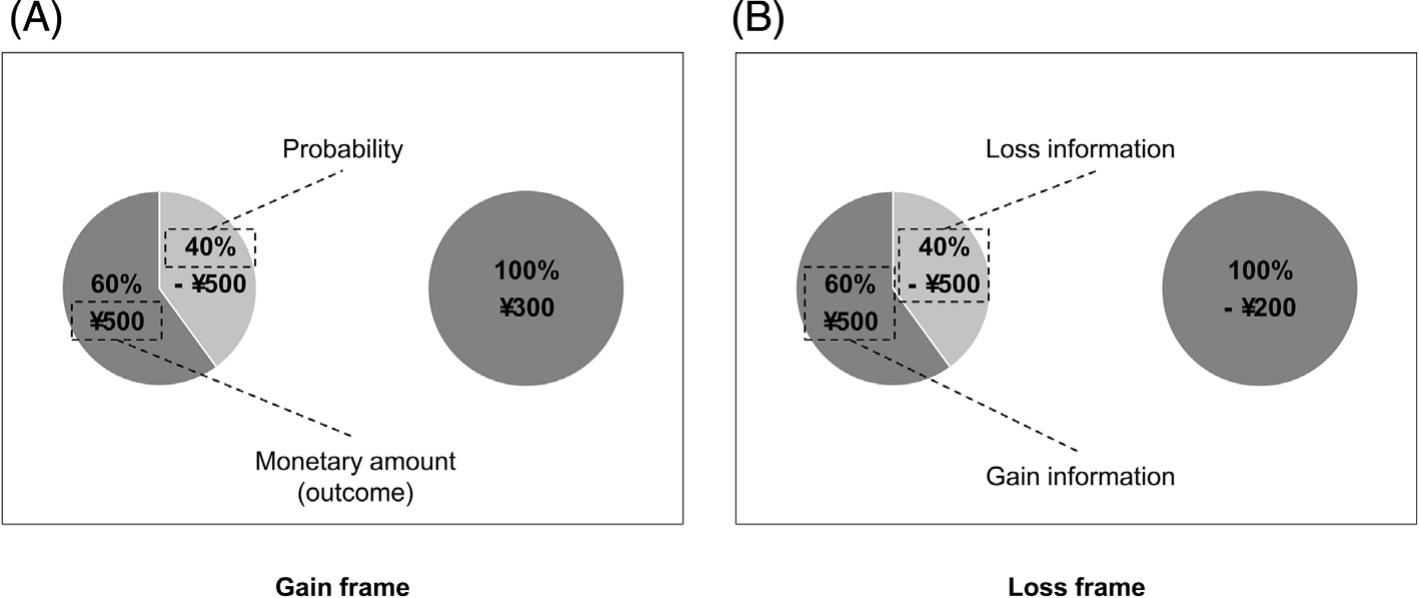
An example of risky choices, with risky and safe options varying in color and choice framing. (A) and (B) show the gain and loss frames for the same choice, respectively. The option on the left is the risky option, which means that decision‐makers have a certain probability of getting all of the amount. The option on the right is the safe option, which means that decision‐makers are sure to keep a portion of the amount. In the risky option, the dark‐grey portion of the pie chart represents gains, and the light‐grey portion represents losses.

The limitations of visual attention prevent us from processing all information simultaneously. Attention is thus directed to a specific object, the choice of which does not occur randomly but is instead influenced by certain factors. Attention has traditionally been divided into bottom‐up and top‐down features, which are driven by stimuli and goals, respectively (Theeuwes, 2010). More recently, researchers have proposed value‐based attention, which is driven by the value of the object attended to (Anderson et al., 2011). In the classical paradigm of risky decision‐making, such as the financial decision‐making task (De Martino et al., 2006), options typically include individuals' gain and loss information, which is composed of monetary amount (outcome) and probability, constituting choices with different risk levels (see Figure 1 for an example). We first summarize how options' bottom‐up, top‐down, and value‐driven features capture attention before choices and change the fixation pattern.

First, although previous studies have tended to emphasize cognitive factors over visual factors, a meta‐analysis has revealed that visual factors (bottom‐up) play a role that is similar to or even more significant than cognitive factors (top‐down) in determining attention (Orquin et al., 2021). Visual factors are usually reflected in the visual features attached to an option, including color, size, location, saliency, and visual format. Studies have shown that the influence of visual factors on bottom‐up attention is revealed through early attention, usually the first fixation (Dambacher et al., 2016; Spitmaan et al., 2019). Options' color, location, and size can capture the first fixation to the more visually salient option, regardless of cognitive factors or option value (Fisher, 2021; Kwak & Huettel, 2018; Roberts et al., 2021; Vanunu et al., 2021). Decision‐makers obtain initial information from these features through early attention, and the corresponding option is processed with higher priority (Kwak & Huettel, 2018; Roberts et al., 2021; Spitmaan et al., 2019).

Second, cognitive factors can significantly change the attentional process. Cognitive factors include individual factors, such as decision‐makers' states, goals, and expectations, as well as high‐level decision context factors, such as cues, tasks, and strategies. Fixation duration epitomizes top‐down attention guided by cognitive factors, reflecting the attentional advantages of one option over another. For example, a higher goal relevance and the expected value strategy rather than the heuristic strategy enable options to receive more fixation duration and counts (Schoemann et al., 2019; Vanunu et al., 2021). Negative factors, such as stress, also impede people's attention to task characteristics (Simonovic et al., 2018). Cues shift attention to the indicated option as an indicative signal, thereby increasing the attentional advantages of relevant information (Cherkasova et al., 2018; Sui et al., 2020).

In addition, cognitive factors influence the pattern of information search. A deliberative state and induced option‐based search strategies prompt decision‐makers to search for information more broadly (Liu et al., 2021; Ludwig et al., 2020; Mittone & Papi, 2020). When participants are instructed that the option they select will be played one time (single‐play task)/one hundred times (multiple‐play task) by the background program, the search pattern used in the single‐play task is similar to the heuristic process (Su et al., 2013). In contrast, the search pattern used in the multiple‐play task is a weighting and adding process described by expected value theory (Su et al., 2013).

Finally, option value affects the whole process of pre‐choice attention for value‐based risky decision‐making. Value‐driven factors include the value of the option itself and learned stimulus–value association (Anderson et al., 2011). High‐value options receive higher attentional priority and prolonged fixation duration (Bourgeois et al., 2017; Fiedler & Glöckner, 2012; Gluth et al., 2020; He & Bhatia, 2023; Vanunu et al., 2021). The last fixation tends to fall on the option with a higher expected value (Liu et al., 2020). Moreover, the information search strategy used during risky decision‐making relates to the expected value of options. If the expected value of the first‐fixated option is high enough, then the information search for choices will stop (Roberts et al., 2021).

In summary, visual, cognitive, and value‐driven factors influence different attentional processes, but all adjust important aspects of attention preceding choices. These results from the experimental manipulation of attention provide the foundation for the causal effect of attention on choices (Krajbich, 2019). However, few theories incorporate the process by which attentional biases arise into attention‐based explanations of risky decision‐making. The effect of the interaction between salient factors on attention when they coexist or conflict with each other still needs further exploration.

## ATTENTIONAL ROLE IN INFORMATION PROCESSING DURING RISKY CHOICES

Studies have revealed that attention‐induced choice biases also occur in risky decision‐making; that is, people tend to choose the option that receives more attention, regardless of the actual value of an option they see (Pachur et al., 2018; Roberts et al., 2021; Stewart et al., 2016). What happens to the captured first fixation and the increased fixation duration that allows attention to play an essential role in risky choices?

Attentional models that incorporate eye‐movement data into the prediction of choices, such as the attentional drift‐diffusion model (aDDM), have been widely used to describe the process of attention in biasing choices and have been extended to the risky decision‐making domain (Ashby, Johnson, et al., 2016; Awh et al., 2012; He & Bhatia, 2023; Krajbich et al., 2010; Krajbich & Rangel, 2011; Molter et al., 2022; Vanunu et al., 2021). According to such models, decision‐making is a process of evidence accumulation. Over time, considerable evidence for each option will be accumulated; once enough evidence has been accumulated for one option (relative to another option), a decision will be made. Attention alters evidence accumulation processes, with first fixation and fixation duration having different effects.

The fixation sequence guides the order of information processing (Kwak & Huettel, 2018; Roberts et al., 2021; Vanunu et al., 2021). The framing effect is amplified when the frame information describing gains or losses is processed earlier (Kwak & Huettel, 2018; Roberts et al., 2021). However, the attentional mechanism of choice is not a primacy effect: first fixation does not affect behavior when choices are made after all options are attended but only when choices are made before all options are fixated (Roberts et al., 2021). This effect seems to suggest that attention does not work merely through first fixation. Vanunu et al. (2021) proposed that bottom‐up attention perceptually codes options, while top‐down attention acts on sampling options (evaluating options as potential outcomes in a way that affects choices). Top‐down attention suppresses the bottom‐up effect and ultimately controls choices when the two are in conflict among options (Vanunu et al., 2021).

First fixation is assumed to have considerable trial noise that does not necessarily enter evidence accumulation in the relevant model (Fisher, 2021; Krajbich et al., 2010; Krajbich & Rangel, 2011). The drift rate (the rate of evidence accumulation towards a specific option) depends on the fixation allocation (Fisher, 2021; He & Bhatia, 2023; Vanunu et al., 2021). Thus, an increased fixation duration means that attentional‐advantage information is sampled more frequently, with its evidence being integrated and accumulated, thereby prompting the relevant option to reach the choice threshold more quickly. In addition, fixation amplifies any option's value, thereby increasing its likelihood of being chosen (Smith & Krajbich, 2019). Sui et al. (2020) verified the causal effect of fixation duration on choice through the gaze‐contingent manipulation paradigm. Gaze‐dependent models that include empirical fixation data provide the best overall account of data when considering the relationship between attention and risky choices (He & Bhatia, 2023; Molter et al., 2022; Thomas et al., 2021).

Notably, risky decision‐making has the nature of multi‐attribute decision‐making, with probabilities and outcomes as different attributes. Therefore, the role of attention should be considered in a multi‐attribute modeling framework. Fisher (2017, 2021) fitted the aDDM based on attention to specific features. Specifically, the weight of unattended attributes will decrease when participants choose between two options, each consisting of a fascinating feature (positive attribute) and an aversive feature (negative attribute). Yang and Krajbich (2023) analysed five two‐option, two‐attribute datasets, including risky choice datasets, and found that the original weight of attributes increased with more attention. However, the two attributes in risky choice datasets chosen by Yang and Krajbich (2023) were simply the two outcomes of each option, without involving different probabilities. Although these studies defined attributes differently, they all indicated that attention to a particular component within an option affects its weight.

In general, such a pattern can be found in existing studies: attention mediates the process by which exogenous information influences risky decision‐making. In this mediation process, attention determines what kind of information is processed and integrated, thereby affecting the rate of evidence accumulation. Several studies have confirmed this mediation effect (Fridman et al., 2018; Sui et al., 2020; van der Laan et al., 2017; Vriens et al., 2020). More evidence is accumulated when fixation duration is longer. However, the relative impact of bottom‐up and top‐down attention needs further clarification. For example, is the bottom‐up effect only on the first fixation, most studies claim that the first fixation does not affect evidence accumulation?

## ATTENTIONAL EFFECT ON RISK PREFERENCE

The greater the level of attention given to a risky option, the higher the probability that that option will be chosen (He & Bhatia, 2023; Molter et al., 2022; Mueller et al., 2022; Sui et al., 2020; Toma et al., 2023; Wang & Liu, 2021). In particular, for risky choices, the choice results are expressed through risk preference, that is, whether people are more risk‐seeking or risk‐averse. Studies have shown that risk preference is related to fixations on specific attributes in options (Ashby et al., 2018; Brandstatter & Korner, 2014; Glickman et al., 2019; Molter et al., 2022; Schoemann et al., 2019). Risky decisions are made by running parallel multiple comparisons between the separate attributes and then integrating the results of the comparisons (Lee et al., 2023). Considering that attention reflects the weight of the specific features described earlier, it is necessary to explain attention to attributes and their impact on risk preference in order to understand the role of attention deeply.

Studies have shown that outcomes receive more fixations than probabilities (Fiedler & Hillenbrand, 2020; He & Bhatia, 2023; Schoemann et al., 2019; Sui et al., 2020). The modeling of probabilities and outcomes by He and Bhatia (2023) indicated that high attention to attributes increases their weight in the drift rate. People will have a higher risk preference when the outcome is given a higher weight by attention, such as insensitivity to probabilities (Cherkasova et al., 2018; Dambacher et al., 2016; Glickman et al., 2019; Mittone & Papi, 2020). High‐risk‐averse individuals tend to focus on probabilities, while low‐risk‐averse individuals or risk‐seekers tend to focus more on outcomes (Clay et al., 2017; Kim et al., 2012; Lee et al., 2023). This may be because the safe option is associated with a higher probability and a lower outcome, while the risky option is associated with a lower probability and a higher outcome (Lee et al., 2023). Thus, decision‐makers prefer options with a higher probability when attention to probability amplifies their weight, thereby showing risk aversion, while those who tend to pay attention to outcomes tend to do the opposite.

The influence of attention to attributes on risk preference can also be represented by the classical preference reversal phenomenon in risky decision‐making; that is, the contradiction between monetary valuations and actual choices. Participants will provide a larger valuation for the option with a larger monetary amount but tend to choose the option with a higher probability (Grether & Plott, 1979; Lichtenstein & Slovic, 1971). Studies have shown that preference reversal is associated with a shift in attention to two attributes in valuation and choice tasks (Alós‐Ferrer et al., 2021; Alós‐Ferrer & Ritschel, 2022; Kim et al., 2012; Zhou et al., 2021). Participants will attend more to monetary amounts during valuation, while they will attend more to probabilities during choice. As a result, there is a bias consistent with attention to attributes in valuation and choice tasks. Furthermore, attention to probabilities and outcomes is interactional; fixations on an option's probability increase subsequent fixations on the outcome of the same option (He & Bhatia, 2023).

In addition to probabilities and outcomes, risky choices sometimes involve gains and losses, which have opposite positive and negative properties for decision‐makers. The distribution of fixations is uneven across gains and losses (Brandstatter & Korner, 2014; Pleskac et al., 2019; Purcell et al., 2021; Zeisberger, 2022). On the one hand, increased fixations on losses predict the decreased subjective value of the corresponding option (Ashby et al., 2018). An attentional bias towards losses may drive a high loss aversion (Clay et al., 2017). On the other hand, paying a higher level of attention to gains significantly increases one's tendency to choose that option (Mueller et al., 2022).

This seems to indicate that fixations on gains or losses may imply opposite (favorable or unfavorable) evidence accumulation processes (Sepulveda et al., 2020). However, giving attention to both positive and negative attributes within an option accumulates evidence about that option equally, but the evidence on the positive attribute accumulates more quickly (Fisher, 2021). Therefore, the effect of attention to gains or losses on evidence accumulation needs to be clarified, especially when gain and loss information appear in the same option (e.g., the two portions of the risky option in Figure 1, or see the 50–50 gambling paradigm in Mueller et al. (2022)).

**FIGURE 2:**
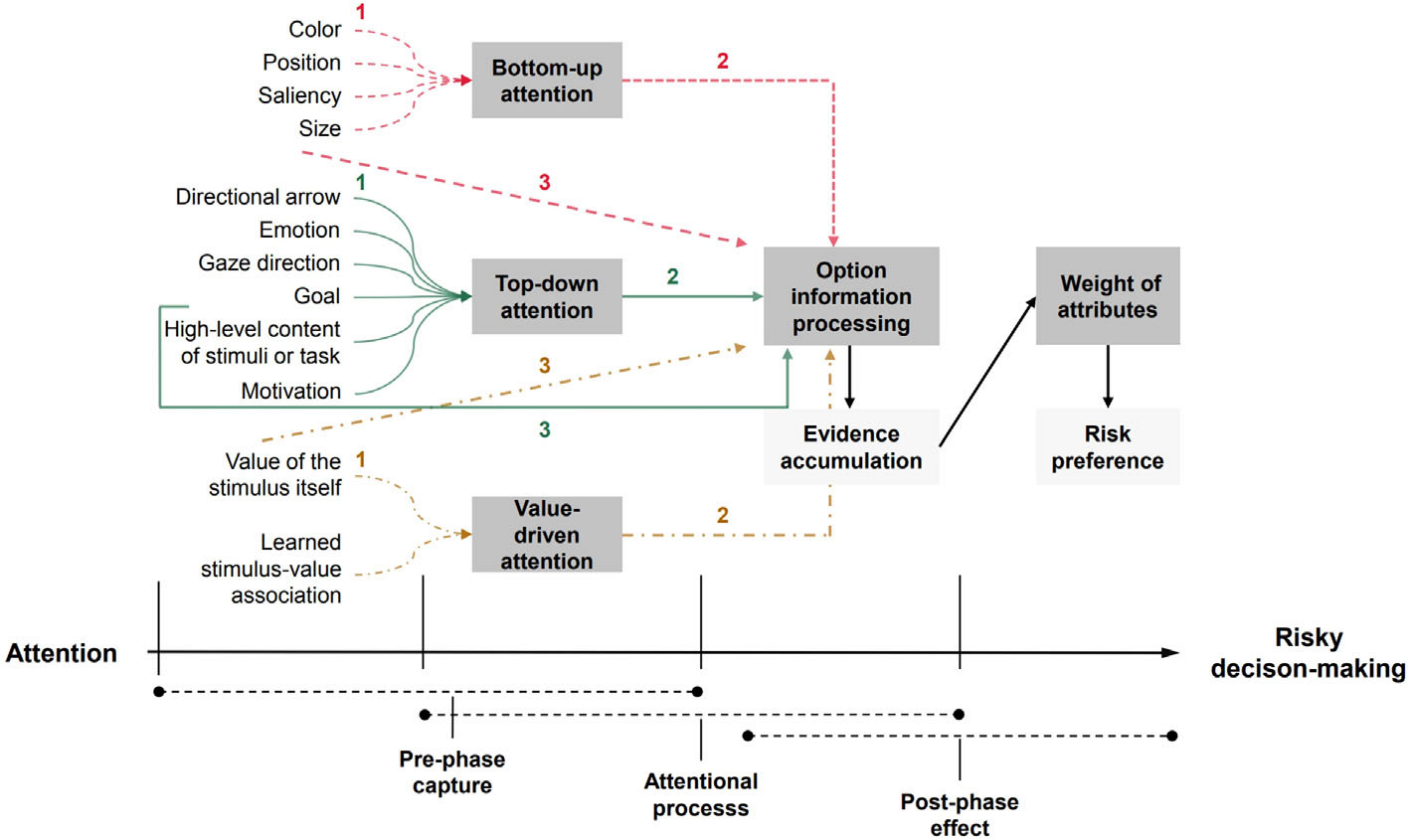
The attentional mechanism of risky decision‐making. The pink dotted lines at the top, the solid green lines in the middle, and the yellow dotted lines at the bottom represent bottom‐up, top‐down, and value‐driven processes, respectively. Path 3 represents the direct impact of these salient factors on risky decision‐making. Paths 1 and 2 show the mediation of attention in this process. Attentional biases change the evidence accumulation of options illustrated by the solid black line, which ultimately influences risk preference through the weight of attributes.

In summary, attention shifts risk preference away from simply multiplying or adding probabilities and outcomes. More fixations on an attribute increase its weight during risky trade‐offs, biasing the final choice towards the option with a higher value on that attribute. An attentional bias towards outcomes corresponds to risk‐seeking, while an attentional bias towards probabilities corresponds to risk‐aversion. These results open up the possibility of shaping risk preference by directing attention during risky decision‐making. However, the effect size and direction of different weights of attributes on risk preference need to be further explored. Here, we briefly summarize risky decision‐making under the attentional mechanism in order to understand the process of risky choices more intuitively (see Figure 2).

## LIMITATIONS AND FUTURE DIRECTIONS IN THIS FIELD

This paper has reviewed recent research that has focused on the relationship between attention and risky decision‐making based on eye‐tracking data. We first summarized the pre‐phase attentional capture before making a choice, involving bottom‐up, top‐down, and value factors in option information. Then, we outlined the biases of information processing under the action of attention, which, combined with the aDDM, govern the evidence accumulation of options. Finally, we focused on the post‐phase effect of attention on risk preference.

However, some problems still need to be solved in investigating the relationship between risky decision‐making and attention. First, more studies are needed to demonstrate the attentional mechanism. One popular claim is that gaze amplifies the subjective value of the attended option (Smith & Krajbich, 2019). However, Sepulveda et al. (2020) combined psychophysics with computational modeling and found that attention does not boost value but rather modulates the integration of goal‐relevant evidence. When the task goal is choosing the best outcome, attention boosts an option's value, while it reduces value when the goal is choosing the worst outcome (Sepulveda et al., 2020). Other explanations might exist for the “higher value, faster RT” relationship (Mormann & Russo, 2021). In addition, the subjective weight of attributes with more fixations increases (Fisher, 2021; He & Bhatia, 2023; Yang & Krajbich, 2023). However, the size and direction of this effect on the drift rate in evidence accumulation are still unknown.

The relationship between attention and value or weights needs to be further explained, which is particularly significant for value‐based multi‐attribute risky decision‐making. Emphasizing the attentional mechanism is not a refutation of traditional economic models; rather, it may provide process‐based explanations for the choice results they describe. For example, attention changes the subjective weight of probabilities and outcomes. Future research may attempt to combine these two streams to illustrate the process of risky decision‐making.

Second, the manipulation and measurement of attention remain problematic. As mentioned above, the direct manipulation of attention is required to verify the causality between attention and risky decision‐making (Krajbich, 2019). However, although a growing number of studies have used eye‐tracking data to predict subsequent choices, the issue of how attention is allocated in the first place under exogenous manipulation needs to be addressed. Many current paradigms for risky decision‐making simplify risky options, which is at odds with the complexity of visual environments in the real world. Therefore, researchers need to clarify the role of salient factors.

Eye‐tracking is widely used to measure attention directly. However, the smallest effect size of attention on choice tends to occur in the most direct attentional manipulation, which may reflect that fixations and attention are not necessarily the same thing (Krajbich, 2019). Participants also use covert attention without direct fixations to help them choose (Perkovic et al., 2023). Two interconnected but separate systems may exist between visual attention and eye movement (Hunt et al., 2019). When focusing on the attentional process during risky decision‐making, more effective measurements should be considered, and multiple methods can be combined, such as mouse‐tracking (Stillman et al., 2020) and virtual reality (Bourgeois et al., 2018).

Finally, decision‐makers' choice patterns may vary in response to stimuli and environments. For example, compared with choosing computer‐based 2D images, people are more risk‐seeking when choosing concrete 3D objects (De Petrillo et al., 2020). Peng et al. (2021) found inconsistent eye‐tracking results for choices in virtual and real‐life environments, highlighting the importance of considering environmental changes when interpreting eye‐tracking data for choices. Researchers need to clarify the reasons for the inconsistencies between stimulus types and experimental environments, not only to demonstrate the actual role of attention in risky decision‐making but also to improve the ecological validity of conclusions.

## CONCLUSIONS

This paper expounds how risky decisions are made under the effect of attention. At the initial stage before choices, visual, cognitive, and value factors related to options direct attention to the salient option, thereby changing the sequence and allocation of attention and information search patterns. In this process, attentional biases affect which information is processed first and integrated continuously. A longer fixation duration implies that the corresponding option accumulates more evidence and reaches the choice threshold faster. For the final choices, attention increases the weight of attributes with attentional advantages, thus biasing risk preference in line with the weight. For example, decision‐makers who are more outcome‐oriented tend to be risk‐seeking. Eye‐tracking provides a basis for exploring the attentional process during risky decision‐making. However, further research is needed to verify the impact of attention on high‐complexity risky decision‐making and to manipulate and measure attention more effectively in order to improve the internal and external validity of the results.

## CONFLICT OF INTEREST STATEMENT

The authors declare that there are no conflicts of interest.
